# Bilingual digital health intervention to improve COVID-19 self-testing intentions among Hispanic adults

**DOI:** 10.3389/fpubh.2025.1662987

**Published:** 2025-12-08

**Authors:** Uchenna Osuagwu Gbugu, Michelle Gil, Bibiana Mancera, Tadesse Abegaz, Gabriel Frietze

**Affiliations:** 1 Interdisciplinary Health Sciences, College of Health Sciences, The University of Texas at El Paso, El Paso, TX, United States; 2 Biological Sciences, College of Health Sciences, The University of Texas at El Paso, El Paso, TX, United States; 3 Department of Pharmaceutical Sciences, School of Pharmacy, The University of Texas at El Paso, El Paso, TX, United States

**Keywords:** digital health intervention (DHI), COVID-19 rapid antigen test, Hispanic, community engaged research, bilingual intervention, digital health

## Abstract

**Introduction:**

Testing remains one of the most effective strategies for reducing the spread of COVID-19. COVID-19 rapid antigen tests are well-suited for at-home use; however, several barriers, including language, hinder the widespread adoption of self-testing. The purpose of the present study is to evaluate the impact of a bilingual digital health intervention on self-efficacy, intentions, and willingness to self-administer a COVID-19 antigen rapid test.

**Methods:**

Participants (*N* = 150) were randomly assigned to one of two experimental conditions: (1) an intervention group who received the bilingual digital health intervention, or (2) a control group who received the standard iHealth® instructions typically included with COVID-19 antigen rapid test kits.

**Results:**

Participants in the bilingual digital health intervention reported significantly higher intentions to self-test for COVID-19 (*M* = 4.26 vs. 3.82), *t*(143) = −2.37, *p* = 0.019, and greater willingness to test themselves (*M* = 4.25 vs. 3.89), *t*(142) = −1.99, *p* = 0.047.

**Conclusion:**

Our findings suggest that the bilingual digital health intervention may have a positive impact on enhancing intentions and willingness to self-administer a COVID-19 antigen rapid test. These findings highlight the potential of culturally and linguistically tailored digital health interventions to reduce barriers and promote access to at-home COVID-19 testing in Hispanic communities. Notably, to help alleviate cost barriers associated with purchasing COVID-19 rapid antigen test kits, the research team collaborated with a highly qualified team of Community Health Workers (CHWs) to disseminate approximately 4,500 free COVID-19 test kits throughout the community.

## Introduction

Coronavirus disease 2019 (COVID-19) is a respiratory infection caused by SARS CoV 2. It is transmitted by airborne particles and droplets released into the air (1). COVID-19 attacks the lungs and respiratory system; therefore, individuals infected with COVID-19 may experience respiratory symptoms that feel quite similar to those of having a cold or flu (2). Many people infected with COVID-19 may experience mild symptoms; however, in others, these symptoms could be severe, resulting in health complications, hospitalization, or even death.

Research shows that people from racial and ethnic minority groups are more likely to be infected with COVID-19. Black and Hispanic people have the highest incidence and death rates due to COVID-19 compared to White people and continue to experience higher rates of infections, hospitalization, and death (3). In June 2020, the Centers for Disease Control (CDC) conveyed that African Americans and Latinx represented 21.8 and 33.8% of the COVID-19 cases, respectively, of the COVID-19 cases in the US (4). As of July 2020, Hispanics were five times more likely to die from COVID-19 compared to White people, and Black people were three times more likely to die from COVID-19 than White people (3). Cumulative COVID-19 age-adjusted mortality rates by race between 2020 and 2022 indicate that Black and Hispanic people experienced a death rate of 466 per 100,000 and 441.9 per 100,000, respectively, compared to 268.5 deaths per 100,000 in White people (3).

One of the most effective methods for reducing the spread of COVID-19 is through testing. To date, there are two main types of tests, namely the Polymerase Chain Reaction (PCR) test and the Antigen test. The PCR test is the ‘gold standard’ for COVID-19 tests. It is a type of nucleic acid amplification test (NAAT), which is more likely to detect the virus than antigen tests (5). PCR tests are often conducted at designated COVID-19 testing locations, clinics, and hospitals, and may take up to 3 days to obtain results. The other COVID-19 diagnostic test is the COVID-19 antigen rapid test, which detects proteins from the virus called antigens. These tests are referred to as rapid tests since they produce results in 10 to 30 min and are more suitable for at-home use. However, since they are not as accurate in detecting the virus as the PCR tests are, the FDA recommends, “2 negative antigen tests for individuals with symptoms or 3 antigen tests for those without symptoms, performed 48 h apart” (5). More recently, COVID-19 antigen rapid tests have been developed for self-administration, which has substantially increased access to COVID-19 testing as individuals can now test themselves in the privacy of their own homes.

There are many different brands of COVID-19 antigen rapid test kits that can be used in home tests to identify positive cases for COVID-19 quickly. During COVID-19 surges, the US Department of Health and Human Services sent out up to four free COVID-19 antigen rapid test kits (the BinaxNOW ® or iHealth® brand) to each American household to help reduce cost and access barriers to testing (6). Despite these efforts to increase access to self-test kits, the experience of the average American has been captured by national headlines such as “*Take- home COVID tests are convenient, but confusing”* (7). Moreover, the lack of bilingual (English and Spanish) COVID-19 rapid self-test instructions magnifies confusion in non-native English speakers, thus reducing the self-efficacy of Spanish-speaking Hispanics to self-administer rapid tests. Importantly, commonly available COVID-19 Antigen Rapid Tests (i.e., iHealth®, etc.) only provide readily available instructions in English, exacerbating Hispanic health disparities. In El Paso County, 72% of the population speaks Spanish at home. Furthermore, many Hispanic families are often comprised of 1^st^, 2^nd^, and 1.5 generations that may not speak English fluently. This is of relevance since Hispanics comprise 18% of the US population and 13% (43 million) of them report speaking Spanish as their primary language at home (8).

To increase the self-efficacy, intention, and willingness of individuals for self-testing using the COVID-19 antigen test, an intervention was required. Therefore, the current study developed and tested a bilingual digital health intervention that can be widely disseminated, impacting not only vulnerable Spanish-speaking populations but English speakers as well. Three specific aims were investigated: (1) to develop a bilingual digital health intervention for COVID-19 antigen rapid tests; (2) to conduct pilot focus groups to evaluate the clarity and acceptability of the intervention; and (3) to recruit community members to test the intervention in a real-world setting. We hypothesized that the bilingual digital health intervention will increase intentions to self-administer a COVID-19 rapid test, willingness to self-administer a COVID-19 rapid test, and self-efficacy to self-administer a COVID-19 rapid test.

## Materials and methods

### Aim 1: procedure

The iHealth® COVID-19 rapid test instructions were transformed into a 6–8th grade reading level, which is recommended for the general public. First, the instructions were reviewed and simplified using basic language. Second, readability statistics were calculated, and the language was adjusted until the scores were lower than 8.0, which is the cutoff for general public reading scores. Specifically, the Flesch–Kincaid reading score is a commonly utilized readability formula used to measure the estimated reading grade level of written scripts. The Flesch–Kincaid examines the length of the sentence based on the number of words in the sentence, and the length of the words based on the number of syllables in the words. A score equal to 6.00 to 6.99 is indicative of a 6th-grade reading level. A score equal to 7.00 to 7.99 is indicative of a 7th-grade reading level. A score equal to 8.00 to 8.99 is indicative of an 8th-grade reading level. Two different scores are calculated to determine the readability of the text: first, the Flesch–Kincaid reading ease score, in which a higher score means a text is easier to read, and a Flesch–Kincaid grade level score, which groups text reading level into different grade level groups based on the US education system. It is important to aim for a reading grade level of 8 or less to ensure the majority of Americans can comprehend the content easily (9). Next, a certified interpreter/translator was hired to convert the 6–8th-grade reading-level English instructions into Spanish. Lastly, bilingual iHealth® COVID-19 rapid test DIY videos were developed to accompany the English and Spanish instructions so that community members would have access to both written and audio/visual instructions. A local media firm (BarracudaPR) was hired to develop the iHealth® COVID-19 rapid test DIY video in both English and Spanish.

### Aim 2: evaluate bilingual digital health intervention

The benign digital health multi-modal and bilingual intervention developed above was evaluated by three focus groups to assess usability and ease of understanding before conducting the randomized controlled trial.

#### Aim 2: participants

Focus groups were conducted with 23 participants. There was a total of three focused (*N* = 3) groups conducted. The first two groups each had eight participants, and the third group had seven participants. The 90-min focus groups evaluate the clarity of the developed iHealth® COVID-19 rapid test written instructions and DIY video. Participants completed a prescreening survey to determine eligibility criteria, which included being 18 years or older, bilingual in both English and Spanish, and residing in El Paso, Texas. Participants were recruited via a social media advertisement that was distributed using Facebook geotargeting with criteria set to match the inclusion criteria (e.g., over the age of 18). Participants who met the inclusion criteria were then invited to participate in the focus group at the scheduled time.

#### Aim 2: measures

Participants were asked 10 questions related to the written instructions and DIY video. The first five questions pertained to “written instructions” for the COVID-19 rapid test. Sample items: *(1) In your opinion, how clear and easy is the COVID-19 rapid test instructional video to understand? (2) If you could improve the instructions, what would you do or change to improve them? (3) How helpful were the instructions in both English and Spanish for understanding how to administer a COVID-19 rapid test? (4) How easily would these written instructions be understood by members of your community (*e.g.*, friends, neighbors, or even community members throughout El Paso)? (5) How easily to you think these instructions will be understood by individuals of all ages… for example, could someone in middle-school understand the instructions as well as an older adult? If not, how could they be improved?* The second set of five questions were related to the COVID-19 rapid test instructional video. Sample items: (1) in your opinion, how clear and easy i*s the COVID-19 rapid test instructional video to understand?* (2) *If you could improve the video, what would you do to improve it?* (3) *How helpful was seeing the video in both English and Spanish for understanding how to administer a COVID-19 rapid test? (4) How easily would these video instructions be understood by members of your community (*e.g.*, friends, neighbors, or even community members throughout El Paso)? Please provide some details. (5) How easily to you think these instructions will be understood by individuals of all ages… for example, could someone in middle-school understand the instructions as well as an older adult? If not, how could it be improved.*

#### Aim 2: procedures

The three focus groups were scheduled to occur on campus at the University of Texas at El Paso (UTEP) campus and temporary parking permits were made available to participants. Upon arrival, participants were instructed to read and sign the Institutional Review Board (IRB) approved informed consent form. Next, participants were assigned a numerical value (to protect their identity) and were asked to view the developed rapid test printed instructions in English and Spanish, as well as the DIY video in English and Spanish. After viewing each, participants were asked probing questions by the facilitator related to the translated materials. Participants were required to review the proposed educational materials and provide feedback. Lastly, participants were thanked and compensated with a $30 electronic gift card, which was sent to their email addresses after participating in the study. A facilitator and a note-taker were present, and focus groups were audio-recorded using the microphone on a laptop. The study was approved by UTEP’s Institutional Review Board (IRB #: 1914943–5).

#### Aim 2: approach to analysis

Content analysis was utilized to analyze audio-recorded focus group data and derive themes. Content analysis is the process of detailing things to make descriptive associations so that themes emerge as complete ideas (10). The themes were coded manually using transcripts from the responses of the participants. Coding of the qualitative data was completed by an expert in qualitative analysis and conducting focus groups. The data from focus groups was utilized to refine the printed instructions and DIY video before being tested in the RCT. The public media firm (BarracudaPR) incorporated feedback from community members to improve the quality of the DIY video interventions.

### Aim 3: participants

A small subset of community members (*N* = 150) were asked to complete a brief 25-min survey assessing self-efficacy in administering the rapid tests. The proposed sample size is a conservative estimate derived from a power analysis using G*Power 3.1. Approximately 128 participants are needed to provide an 80% chance of correctly rejecting the null hypothesis that the treatment and control groups are the same. Approximately 110 participants are needed to provide an 80% chance of correctly rejecting the null hypothesis that the correlation coefficient equals zero between self-efficacy and willingness to administer a COVID-19 self-test. Thus, 150 participants are a conservative estimate that accounts for missing data or attrition.

#### Aim 3: measures

As part of the data collection process, common data elements from the National Institute of Health (NIH) Rapid Acceleration of Diagnostics–Underserved Population (RADx-UP) were utilized. The NIH developed these data elements to ensure consistency in data collection and simplify data analysis, and is an initiative to help implement COVID-19 data testing and better understand the impact of the pandemic, such as the outcomes, disparities, and possible solutions. The measures are provided within the supplementary materials.

#### Aim 3: procedure

Participants were prescreened to determine eligibility criteria, which included being 18 years of age or older, residing in the border region (e.g., El Paso, TX), and speaking English or Spanish. One hundred and fifty community members were recruited to complete a brief 25-min survey assessing self-efficacy in administering the rapid tests. Additionally, participants were randomly assigned to one of two conditions using block randomization: (1) a treatment condition in which participants completed the self-efficacy assessment to administer the COVID-19 rapid test after viewing the developed bilingual instructions and video, and (2) a control condition in which participants completed the self-efficacy assessment to administer the COVID-19 rapid test after viewing the standard iHealth® COVID-19 rapid test instructions that are provided from the supplier. Participants completed the survey on-site (e.g., at a tabling event) using a project iPad. Informed consent was obtained from all subjects in the study. All methods were carried out in accordance with relevant guidelines and regulations by UTEPs IRB (#: 1914943–5).

Participants were recruited from local health clinics, food banks, and tabling events in the community. Notably, Community Health Workers (CHWs) assisted in recruiting participants at tabling events. CHWs or Promotores de Salud are public health workers at the forefront of serving their communities. They are trusted members of their community with a great understanding of their community (11). They typically share the same language, ethnicity, and reside in the same communities. As trusted members of their community, they help to bridge the gap between their community and healthcare providers by: (1) aiding in health education to promote health behavior and disease prevention, (2) facilitating access to appropriate care and resources, and (3) advocating for their community members. The work of CHWs have been shown to improve access to healthcare services, enhance communication and better understanding between community members and health providers, and improve adherence to health recommendations, leading to an improved overall health of their community (12). The team of trusted CHWs was expected to increase community participation in the RCT.

#### Aim 3: approach to analysis

All statistical analyses were conducted using SPSS version 29.0.2.0. Fundamental descriptive statistics were conducted for all the demographic variables in the experimental and control groups. Means and standard deviations were calculated for continuous variables and percentages for categorical variables. Composite scores were created for variables that included multiple items. A Cronback alpha was calculated for each of these variables and if the score was less than 0.70, the items were not combined. Independent samples *t*-tests were conducted to compare the control and treatment group on each outcome variable. A Bonferroni correction was applied (alpha = ^0.05^/_3_ = 0.0167) to one of the outcome variables (perceived ease of understanding written instructions), given that the Cronback alpha for the three-items did not meet the 0.70 threshold. Five exploratory hierarchical linear regression models controlled for age, sex, education, and language (Spanish = 1, English = 2) while examining group differences in: (1) self-efficacy in administering COVID-19 rapid tests, (2) future intentions to self-administer a COVID-19 rapid test, (3) intentions to administer a COVID-19 test to family member, (4) willingness to test self for COVID-19, and (5) willingness to test a family member for COVID-19. Specifically, in Step 1 of each regression model the following variables were entered: age, sex, education, and language. In Step 2 of the regression models, the experimental condition (1 = control, 2 = intervention) was entered. Lastly, correlation analyses were conducted to examine associations between continuous variables. There were less than or equal to 5.9% missing data for each of the variables of interest, therefore, multiple imputation was not conducted and for each analysis we used a case-wise deletion approach.

## Results

### Aim 1: results

A Flesch–Kincaid reading score of 7.9 grade level was obtained along with a Flesch–Kincaid reading ease score of 70.9. The newly translated English instructions were then translated into Spanish instructions using a certified translator. The contracted local media firm (BarracudaPR) developed two iHealth® COVID-19 rapid test DIY videos, one in English and another in Spanish.

### Aim 2: results

The focus groups revealed two areas of opportunity for improvement: (1) Language barrier—not having instruction in Spanish and at a reading level that could be easily understood, and (2) Combination—visualization of the demonstration with text and voice (spoken word) were very helpful, making it easy for anyone to understand and conduct the rapid test given the clarity of the video.

Language as a barrier was a recurring theme throughout all three focus groups. Participants were asked questions to assess the clarity of the written instructions, for example, “In your opinion, how clear and easy are the COVID-19 rapid antigen test instructions?” One participant stated the following: “The words that they use should be words that people use (common language) that are more commonly used.” The second participant stated, “…the words need to be simpler to better understand the instructions. Simple and short. The easier the better.” Another participant stated, “I would like the instructions to be in Spanish because you could understand them and you will not make a mistake.” The majority of participants expressed similar sentiments.

The second theme that emerged among all of the study participants was the perceived benefit from the combination of visual and spoken words through the DIY video. Participants were asked, “In your opinion, how clear and easy is the video to understand the instructional COVID-19?” One participant responded, “It was very clear. It was to the point. It showed you how to do it, step by step. It did not skip any information. It was very good. She was giving the instructions while she was doing it in the video.” Another question about the video asked, “How easily do you think these instructions will be understood by members of your community, your friends, neighbors?” A participant responded, “Personally, feeling like there is someone with you guiding you, you feel more confident.” Another participant responded, “In another language other than your own, it’s 95%, but in your own language, it’s 100%!”

### Aim 3: results

A total of 152 participants (*M*_age_ = 48.50, *SD* = 14.92) were recruited to participate in the study. Most of the sample (81.6%) reported that their biological sex was female and also identified as women (80.3%). The sample participants were reported to be predominantly Hispanic (95.4%). Approximately a third of the sample (33.6%; *n* = 51) of the sample completed the survey in English, and 66.4% (*n* = 101) completed the survey in Spanish. More than three-quarters of the sample (77.6%; *n* = 118) reported that Spanish was their preferred language spoken at home. 53.3% (*n* = 81) indicated that they read, understand, and speak English at home compared to 92.1% (*n* = 140) for Spanish. Approximately two-thirds (67%) of the sample reported that their annual income was below $35,000. A quarter of the sample reported having completed a 12th-grade or lower education level with no diploma, 31.6% completed high school or have a GED, and 9.9% reported having attained a bachelor’s degree. About half (52.6%) of the study participants reported being currently employed, with 8.6% unemployed and looking for work, and 9.9% retired. Just over a quarter of the study participants reported not having health insurance (26.3%). See Table 1.

**Table 1:** Demographic and background information (*N* = 152).

Variable	Total responses	Percentage of responses
Sex assigned at birth		
Male	27	17.8%
Female	124	81.6%
Prefer not to answer	1	0.7%
Ethnicity		
Non-Hispanic	4	2.6%
Hispanic	145	95.4%
Prefer not to answer	3	2%
Missing	0	0%
Race		
American Indian or Alaskan Native	0	0%
Black or African American	0	0%
Asian	0	0%
Native Hawaiian or Other Pacific Islander	0	0%
White	125	82.2%
Some other race	14	9.2%
Prefer not to answer	12	7.9%
Primary/preferred language		
English	32	21.1%
Spanish	118	77.6%
Missing	2	1.3%
Household income		
Less than $15,000	47	30.9%
$15,000–$19,999	18	11.8%
$20,000–$24,999	21	13.8%
$25,000–$34,999	16	10.5%
$35,000–$49,999	12	7.9%
$50,000–$74,999	13	8.6%
$75,000–$99,999	4	2.6%
$100,000 and above	2	1.3%
Prefer not to answer	18	11.8%
Missing	1	0.7%

#### Self-efficacy in administering a COVID-19 test

An independent samples *t*-test was conducted to examine the self-efficacy in administering a COVID-19 test. To do this, the following three variables were combined to create a self-efficacy variable: I believe the COVID-19 rapid test was easy to administer, I felt confident to administer the COVID-19 rapid test to myself, and I would feel confident administering the COVID-19 rapid test to someone else. There was no statistically significant difference between the treatment group who received the bilingual instructions (*M* = 3.93, *SD* = 1.27) and the control group who received the standard iHealth® Instructions (*M* = 3.54, *SD* = 1.17), *t*(141) = −1.89, *p* = 0.06, 95% CI [−0.79, 0.017]. However, one of the variables ease of self-administering the COVID-19 rapid test resulted in a statistically significant difference between the treatment group who received the bilingual instructions (*M* = 3.88, *SD* = 1.41) and the control group who received the standard iHealth® Instructions (*M* = 3.38, *SD* = 1.36), *t*(145) = −2.21, *p* = 0.029, 95% CI [−0.96, −0.05].

An exploratory hierarchical regression model was used to assess impact of the intervention on self-efficacy in administering a COVID-19 test, after controlling for age, sex, education, and language. Preliminary analyses ensured no serious violations of the assumption of normality, linearity, multicollinearity, and homoscedasticity. The model was not statistically significant (*p* = 0.322).

#### Perceived ease of understanding the written instructions

To assess perceived ease of understanding written instructions, an independent *t*-test was conducted to analyze three variables separately and a Bonferroni correction was applied (alpha = 0^.05^/_3_ = 0.0167). After applying the Bonferroni correction, none of the *t-*tests were statistically significant. Specifically, when participants were asked if they believe the COVID-19 rapid test instructions was easy to understand, there was no difference between the treatment group who received the bilingual instructions (*M* = 3.93, *SD* = 1.40) and the control group who received the standard iHealth® Instructions, *p* = 0.027. Additionally, when participants were asked if they believe the COVID-19 Rapid test written instructions had easy to understand language, there was no difference (*p* = 0.076). Lastly, when asked if they believe the COVID-19 written instructions had confusing language, there was no difference (*p* = 0.284).

#### Intention to test self for COVID-19

An independent samples *t*-test was conducted to examine the intention to self-test for COVID-19. A statistically significant difference emerged between the treatment group, who received the bilingual instructions (*M* = 4.26, *SD* = 1.01), and the control group, who received the standard iHealth® Instructions (*M* = 3.82, *SD* = 1.24), *t*(143) = −2.37, *p* = 0.019, 95% CI [−0.82, −0.07].

An exploratory hierarchical regression model was used to assess impact of the intervention on intention to test self for COVID-19, after controlling for age, sex, education, and language. Preliminary analyses ensured no serious violations of the assumption of normality, linearity, multicollinearity, and homoscedasticity. Age, sex, education, and language were entered at Step 1, explaining 6.9% of the variance in intentions to test self. After entry of the experimental conditions at Step 2, the total variance explained by the model was 9.6%, *F* (5, 136) = 2.88, *p* = 0.017. The intervention explained an additional 3% of the variance in intentions to test self, after controlling for age, sex, education, and language, R squared change = 0.03, *F* change (1, 136) = 3.99, *p* = 0.048. In the final model, only two variables were statistically significant, age (beta = −0.22, *p* = 0.009) and the experimental condition (beta = 0.17, *p* = 0.048).

#### Intention to test family for COVID-19

An independent samples *t*-test was conducted to examine the intention to test family members for COVID-19. A statistically significant difference emerged between the treatment group, who received the bilingual instructions (*M* = 4.23, *SD* = 1.02), and the control group, who received the standard iHealth® Instructions (*M* = 3.75, *SD* = 1.28), *t*(145) = −2.53, *p* = 0.012, 95% CI [−0.86, −0.11].

An exploratory hierarchical regression model was used to assess impact of the intervention on intention to test family for COVID-19, after controlling for age, sex, education, and language. Preliminary analyses ensured no serious violations of the assumption of normality, linearity, multicollinearity, and homoscedasticity. Age, sex, education, and language were entered at Step 1, explaining 8.3% of the variance in intentions to test family. After entry of the experimental conditions at Step 2, the total variance explained by the model was 11%, *F* (5, 138) = 3.42, *p* = 0.006. The intervention explained an additional 2.8% of the variance in intentions to test family, after controlling for age, sex, education, and language, R squared change = 0.028, *F* change (1, 138) = 4.31, *p* = 0.040. In the final model, only two variables were statistically significant, age (beta = −0.26, *p* = 0.002) and the experimental condition (beta = 0.17, *p* = 0.04).

#### Willingness to test self for COVID-19

An independent samples *t*-test was conducted to examine the willingness to self-test for COVID-19. A statistically significant difference emerged between the treatment group, who received the bilingual instructions (*M* = 4.25, *SD* = 1.00) and the control group, who received the standard iHealth® Instructions (*M* = 3.89, *SD* = 1.13), *t*(142) = −1.999, *p* = 0.047, 95% CI [−0.71, −0.004].

An exploratory hierarchical regression model was used to assess impact of the intervention on willingness to test self for COVID-19, after controlling for age, sex, education, and language. Preliminary analyses ensured no serious violations of the assumption of normality, linearity, multicollinearity, and homoscedasticity. Age, sex, education, and language were entered at Step 1, explaining 8.3% of the variance in willingness to test self. After entry of the experimental conditions at Step 2, the total variance explained by the model was 9.9%, *F* (5, 135) = 3.21, *p* = 0.015. The intervention explained an additional 1.5% of the variance in willingness to test self, after controlling for age, sex, education, and language, R squared change = 0.015, *F* change (1, 135) = 2.27, *p* = 0.134. In the final model, only one variable was statistically significant, age (beta = −0.26, *p* = 0.002).

#### Willingness to test family for COVID-19

An independent samples *t*-test was conducted to examine the willingness to test a family member for COVID-19. There was no difference between the treatment group who received the bilingual instructions (*M* = 4.21, *SD* = 0.94) and the control group who received the standard iHealth® Instructions (*M* = 3.90, *SD* = 1.13), *t*(142) = −0.813, *p* = 0.072, 95% CI [−0.66, 0.03].

An exploratory hierarchical regression model was used to assess impact of the intervention on willingness to test family for COVID-19, after controlling for age, sex, education, and language. Preliminary analyses ensured no serious violations of the assumption of normality, linearity, multicollinearity, and homoscedasticity. Age, sex, education, and language were entered at Step 1, explaining 7.1% of the variance in willingness to test family. After entry of the experimental conditions at Step 2, the total variance explained by the model was 8.3%, *F* (5, 135) = 2.46, *p* = 0.036. The intervention explained an additional 1.2% of the variance in willingness to test family, after controlling for age, sex, education, and language, R squared change = 0.012, *F* change (1, 135) = 1.81, *p* = 0.181. In the final model, only one variable was statistically significant, age (beta = −0.24, *p* = 0.005).

#### Perceived benefit of audio and visual intervention

Correlational analyses revealed that ease of understanding the video was positively correlated with the perceived benefit of the video in self-administering a COVID-19 test (*r* = 0.81, *p* < 0.001), the perceived benefit of the video to others administering a COVID-19 test (*r* = 0.82, *p* < 0.001), willingness to recommend the video to others (*r* = 0.81, *p* < 0.001), and willingness to purchase a COVID-19 test if they had access to the video (*r* = 0.68, *p* < 0.001). See Table 2 for correlation analyses.

**Table 2:** Correlates of ease of understanding and perceived benefit of written and video instructions.

	1	2	3	4	5	6	7	8
Ease of understanding written instructions	-							
Written instructions in easy to understand language.	0.93**							
Written instructions are in confusing language	0.06	0.05						
Video instructions are easy to understand	0.77**	0.76**	0.05					
Perceived benefit of video to self-administer test	0.77**	0.77**	0.77**	0.81**				
Perceived benefit of video to others to test	0.65**	0.65**	0.06	0.82**	0.85**			
Willingness to recommend video to others	0.71**	0.71**	0.01	0.81**	0.86**	0.95**		
Willingness to purchase COVID-19 test if I had access to video	0.61**	0.62**	0.08	0.68**	0.78**	0.84**	0.88**	
Mean (SD)	3.69 (1.37)	3.73 (1.34)	2.50 (1.28)	4.00 (1.27)	4.05 (1.22)	4.10 (1.13)	4.19 (1.04)	3.99 (1.09)
*N*	84	84	81	84	84	84	84	84

Correlations are reported using Pearson Correlations. *p < 0.05. **p < 0.0.01.

## Discussion

Several important findings emerged in the current study, emphasizing the importance of language in self-efficacy, intentions, and willingness to administer a COVID-19 rapid test. We hypothesized that the bilingual digital health intervention would increase self-efficacy, willingness, and intentions to administer a COVID-19 rapid test. Willingness and intentions to self-administer a COVID-19 rapid test were higher in the group who received the bilingual digital health intervention in comparison to the control group. However, the current study did not detect statistically significant differences in self-efficacy to administer a COVID-19 rapid test. Studies have shown that visual aids such as pictograms and videos significantly improved health literacy outcomes by increasing adherence to medications and understanding of health information (13), highlighting the benefits of instructional videos for improving health outcomes. Moreover, research suggests that being exposed to videos as a health resource may be effective at improving knowledge or adherence. For example, in a study by Ratri et al. (14), which sought to understand the impact of video instructions on reinforcing insulin therapy in patients with type 2 diabetes, patients were given a pre-test to assess their knowledge and attitudes. Patients subsequently watched a video providing information about type 2 diabetes mellitus and insulin therapy. Patients were then given a post-test questionnaire to assess their knowledge after watching the video. Results suggest that patients’ knowledge and attitudes about insulin were improved after watching the video (14). Public health researchers should continue to explore the impact of utilizing instructional videos and other digital health interventions to help prevent or self-manage various disease states and illnesses. For example, Diabetes Self-Management Education and Support (DSMES) programs might benefit from the use of instructional videos to help patients living with diabetes self-manage their diabetes.

Focus groups are effective for obtaining valuable information related to the development of questionnaires and exploring diverse opinions (15). Trenkner et al. reports that focus groups can be useful for evaluating the clarity and user friendliness of educational materials. In the current study, focus groups were used to gain feedback and evaluate the usability and friendliness of the translated bilingual written instructions and video. Having feedback from the community was an important step toward ensuring that the written instructions and DIY videos developed from the current study were easy to understand and included clear language. Our focus groups also revealed that participants greatly appreciated having the COVID-19 rapid test instructions accessible in their preferred language. These findings highlight the potential of culturally and linguistically tailored digital health interventions to reduce barriers and promote access to at-home COVID-19 testing in Hispanic communities.

Findings from the current study demonstrate the effectiveness of a bilingual digital health intervention at improving intentions to self-administer a COVID-19 rapid test. A goal of this study was to reduce the barriers that individuals face when trying to test themselves for COVID-19 through the development of our bilingual digital health intervention. Developing an effective bilingual digital health intervention was important for several reasons. First, when patients understand how to properly administer their tests, it increases the probability of getting an accurate diagnosis. When instructions are not properly followed, the likelihood of obtaining a false positive or false negative result is increased, which can lead to misdiagnosis. Second, it prevents potentially costly and dangerous repercussions. Communicating health instructions in a language the patient understands can prevent the risk of unknowingly spreading COVID-19 to others due to assuming they have a false negative result. Third, educating patients on how to accurately test themselves for COVID-19 in a language they can easily understand can lead to timely diagnosis, isolation, and treatment. This can reduce the spread of COVID-19 infection and also potentially serious side effects of COVID-19. Fourth, communicating with patients in a language they can understand empowers the patient to feel confident, engaged, and responsible for their health care. It builds trust between me and the patient, thereby allowing them to be receptive to public health recommendations concerning COVID-19. Notably, to help alleviate cost barriers associated with purchasing COVID-19 rapid antigen test kits, the research team collaborated with a highly qualified team of Community Health Workers (CHWs) to disseminate approximately 4,500 free COVID-19 test kits throughout the community at venues including food pantries, local clinics, high schools, libraries, and community centers.

## Limitations

There were several limitations pertaining to this study. The first limitation is that self-reported measures were used which can lead to response bias. Another limitation of the current study concerns the generalizability of the findings given that the study participants were mostly female (81.6%), of Hispanic origin (95.4%), Spanish speaking, and resided in El Paso, Texas. A different quantitative study using a larger and more diverse population could help improve the generalizability of the study. Additionally, the current study did not include objective or behavioral measures which could help identify whether participant’s intentions actually contribute to the behavior of self-administering a COVID-19 rapid antigen test.

## Future directions

Although the current study focused on translating written instructions for the COVID-19 test kit, future studies should investigate the impact of having written and or video instructions available in Spanish for other at-home test kits available on the market, such as Flu, glucose, pregnancy test, and drug tests. Future studies should also include survey questions that assess the participant’s trust in the test kit, as this is a key factor in understanding perceptions of the COVID-19 test kit and any fears or trust issues that need to be addressed.

## Data Availability

The raw data supporting the conclusions of this article will be made available by the authors, without undue reservation.
