# Supplementary material for: Bilingual digital health intervention to improve COVID-19 self-testing intentions among Hispanic adults
Source: Front Public Health. 2025 Dec 8;13:1662987. doi: 10.3389/fpubh.2025.1662987 (PMC12722827; doi:10.3389/fpubh.2025.1662987)
Supplement: Supplementary file 1 [file Table_1.docx]

Supplementary Materials. Description of the measures included in the study.

| **Variable Name** | **Description** | **Sample Item(s)** |
| --- | --- | --- |
| Sociodemographic | A 15-item survey assessed basic demographics such as age, gender, ethnicity, pregnancy status and related information | “Please indicate if you are Hispanic, Latino or Spanish origin.” Response options included (1) No, not of Hispanic, Latino, or Spanish origin, (2) Yes, of Hispanic, Latino, or Spanish origin, and (3) Prefer not to answer. These items were sourced from the phoenixtoolkit (PhenX Toolkit 2024). |
| Housing Employment and Insurance | A 24-item survey assessed housing, employment, and insurance information such as current employment status, having a place to stay/live, having clean water to drink, getting enough food to eat, and related information. | “Did you lose health coverage because of COVID-19 pandemic”. Response options included (1) Yes, (0) No, (98) prefer not to answer, (99) Don’t know. These items were sourced from the Phoenixtoolkit (PhenX Toolkit 2024). |
| Work, PPE and Distancing | A 3-item survey assessed employee access to necessary facilities to wash, access to necessary personal protective equipment, and requirement for close contact with others. | “In your workplace, do you have access to necessary personal protective equipment”. Response options included (1) Yes, all the time, (2) Yes, most of the time, (3) Some of the time, (4) Rarely, (5) Not at all, (6) Not applicable, (99) Prefer not to answer. These items were sourced from the phoenixtoolkit (PhenX Toolkit 2024). |
| Medical History | A single item assesses underlying health conditions. | “Do you have any of these following health conditions? (select all that apply). “Ten response options were provided: “immunocompromised condition”, “autoimmune disease”, “hypertension (HTN, high blood pressure)”, “Diabetes”, Chronic kidney disease (CKD)”, “Cancer diagnosis and/or treatment within the past 12 months”, “cardiovascular disease (CVD or heart disease)”, “asthma”, “Chronic obstructive pulmonary disease (COPD)”, “other chronic lung disease”, “sickle cell anemia”, “depression”, “alcohol or substance use disorder”, “intravenous drug use”, “other mental health disorder”, “other chronic condition”. Response options included (1) Yes, (2) No, (99) Prefer not to answer. These items were sourced from the phoenixtoolkit (PhenX Toolkit 2024). |
| Health Status | A 4-item survey assessed the health status of participants. | “Would you say your health in general is excellent, very good, good, fair, or poor? ” Response options included (1) Excellent, (2) Very good, (3) Good, (4) Fair, (5) Poor, (6) Prefer not to answer, (7) Don’t know. These items were sourced from the phoenixtoolkit (PhenX Toolkit 2024). |
| Disability | An 8-item survey assessed difficulty dressing, difficulty walking, difficulty hearing, and related information. | “Are you deaf, or do you have serious difficulty hearing”. Response options included (1) Yes, (2) No, (99) Prefer not to answer. These items were sourced from the phoenixtoolkit (PhenX Toolkit 2024). |
| Self-efficacy in Administering COVID-19 Rapid Tests | A 3-item survey assessed ease of administering the COVID-19 Rapid test. The Cronbach alpha for the three items was equal to 0.889 in the current study. | “I believe the COVID-19 Rapid Test was easy to administer”. Response options ranged from (1) strongly disagree, to (5) strongly agree and (99) for prefer not to answer. |
|  |  |  |
| Perceived ease of understanding written instructions and language | A 3-item survey assessed the ease of understanding the written instructions. The Cronbach alpha for the three items was equal to 0.540 in the current study. These items were analyzed independently as 3 separate items. | “I believe the COVID-19 Rapid Test written instructions were easy to understand”. Response options ranged from (1) strongly disagree, to (5) strongly agree and (99) for prefer not to answer. |
| Perceived Ease of understanding the video | A 3-item survey assessed the ease of understanding the video. The Cronbach alpha for the three items was equal to 0.933 in the current study. | “I believe the COVID-19 Rapid Test video was easy to understand”. Response options ranged from (1) strongly disagree, to (5) strongly agree and (99) for prefer not to answer. |
| Perceived benefit of the video | A 1-item survey assessed the perceived benefit of the video. | “I would be willing to purchase a COVID-19 test in the future if I had access to the video. Response options ranged from (1) strongly disagree, to (5) strongly agree and (99) for prefer not to answer. |
| Future Intentions to Self-Administer COVID-19 test  Future Intentions to administer COVID-19 test to family member | A 3-item survey assessed intentions to self-test for COVID-19 in the future. The Cronbach alpha for the three items was equal to 0.978 in the current study  A 2-item survey assessed intentions to test family for COVID-19 in the future. The Cronbach alpha for the two items was equal to 0.974 in the current study | “I intend to test myself for COVID-19 in the future if I am feeling COVID-19 symptoms such as a running nose, fever, or coughing.” Response options ranged from (1) strongly disagree, to (5) strongly agree and (99) for prefer not to answer.  “I intend to test my family member for COVID-19 in the future if I am feeling COVID-19 symptoms such as a running nose, fever, or coughing.” Response options ranged from (1) strongly disagree, to (5) strongly agree and (99) for prefer not to answer. |
| Willingness to test self for COVID-19  Willingness to test family member for COVID-19 | A 3-item survey assessed willingness to test self for COVID-19 in the future. The Cronbach alpha for the three items was equal to 0.967 in the current study  A 2-item survey assessed willingness to test self for COVID-19 in the future. The Cronbach alpha for the two items was equal to 0.979 in the current study. | “I am willing to test myself for COVID-19 in the future if exposed to someone who has tested positive for COVID-19”. Response options ranged from (1) strongly disagree, to (5) strongly agree and (99) for prefer not to answer.  “I am willing to test my family member for COVID-19 in the future if I am feeling COVID-19 symptoms such as a running nose, fever, or coughing.” Response options ranged from (1) strongly disagree, to (5) strongly agree and (99) for prefer not to answer. |
| Vaccine Acceptance | A 5-item survey assessed the vaccine history for flu and COVID-19. | “Have you ever received a flu vaccination”. Response options included (1) Yes, (2) No, (98) Don’t know, (99) Prefer not to answer. These items were sourced from the phoenixtoolkit (PhenX Toolkit 2024). |
| Testing | A 12-item survey assessed the COVID-19 testing history, ease, and access to testing and related information. | “How were you tested for your most recent test”. Response options included (1) Nasal swab, (2) Throat swab, (3) Blood sample, (4) Saliva, (99) Prefer not to answer. These items were sourced from the phoenixtoolkit (PhenX Toolkit 2024). |
| Alcohol and Tobacco | A 5-item survey assessed the smoking and alcohol habits of the participants. | “Do you now smoke cigarettes”. Response options included (4) Every day, (3) Some days, (2) Rarely, (1) Not at all, (98) Prefer not to answer, (99) Don’t know. These items were sourced from the phoenixtoolkit (PhenX Toolkit 2024). |
| Attention Check | A single item was used as an attention check. | “If you are paying attention please select the color “red” below”. Response items included (1) Green, (2) Yellow, (3) Blue, (4) Red, (5) Orange. |
| Manipulation Check | A single item was used as a manipulation check to ensure that the participant viewed the entire video. | “Did you watch the entire video?” Response options included: (1) Yes, (2) No, (please ask a team member for assistance). Participants who selected response option “No” were redirected back to the video and informed to ask a team member for assistance to view the video. This approach ensured that all participants viewed the entire COVID-19 DIY video. Only two participants reported that they did not watch the video. |
| Identity | A 14-item survey assessed the identity of the participants such as name, address, phone number, and related information. | Sample item: “Preferred method of contact”. Response items included (1) Mobile phone, (2) Home phone, (3) Other phone, (4) Personal email, (5) Other email, (99) Prefer not to answer. These items were sourced from the Phoenixtoolkit (PhenX Toolkit 2024). |
| Symptoms | A single item assessed symptoms experienced during the past week. | “Have you had any of these symptoms during the past week? Fever or chills, cough, shortness of breath or difficulty breathing, lack of energy or general tired feeling, muscle or body aches, headache, new loss of taste or smell, sore throat, congestion, or runny nose, feeling sick to your stomach or vomiting, diarrhea, abdominal pain, skin rash, other”. Response options included (1) Yes, (0) No, (98) Don’t know, (99) Prefer not to answer. These items were sourced from the Phoenixtoolkit (PhenX Toolkit 2024). |
| COVID-19 Test Result | A single item assessed result of COVID-19 Test | “What was the result of your COVID-19 test? (Please look at the test you administered to yourself)”. Response options includes: (1) Positive: Both a C and T line are present, (2) Negative: Only the C line is present, (3) Invalid test: No lines or only a T line is present. |
| Confidence to report COVID-19 Test Result | A 2-item survey assessed confidence to report COVID-19 test results. | “I feel confident that I would know how to report my COVID-19 test results using the iHealth^®^ phone application”. Response options ranged from (1) strongly disagree, to (5) strongly agree and (99) for prefer not to answer. |
| Willingness to report a positive COVID-19 test result | A 3-item survey assessed willingness to report their COVID-19 test result if it was positive. | If I tested positive, I would be willing to report my COVID-19 test results using the iHealth^®^ phone application. Response options ranged from (1) strongly disagree, to (5) strongly agree and (99) for prefer not to answer. |
| Intention to report a positive COVID-19 test result. | A 2-item survey assessed intentions to report a positive COVID-19 test result. | “If I tested positive, I intend to report my COVID-19 test result to my healthcare provider”. Response options ranged from (1) strongly disagree, to (5) strongly agree and (99) for prefer not to answer. |
